# Tissue tropism and viral levels of Acheta domesticus densovirus throughout the house cricket production

**DOI:** 10.1016/j.cris.2025.100121

**Published:** 2025-12-20

**Authors:** Jozsef Takacs, Astrid Bryon, Annette B. Jensen, Joop J.A. van Loon, Vera I.D. Ros

**Affiliations:** a Wageningen University and Research, Laboratory of Virology, Droevendaalsesteeg 1, 6708PB Wageningen, the Netherlands; b University of Copenhagen, Department of Plant and Environmental Sciences, Thorvaldsensvej 40, DK-1871 Copenhagen, Denmark; c Wageningen University and Research, Laboratory of Entomology, Droevendaalsesteeg 1, 6708PB Wageningen, the Netherlands

**Keywords:** *Acheta domesticus*, Acheta domesticus densovirus, House cricket, Insect production, Insects as food and feed, Cricket viruses, Entomopathogenic viruses, Mass-rearing

## Abstract

The house cricket, *Acheta domesticus,* is commonly reared for food and feed purposes and is often infected with the Acheta domesticus densovirus (AdDV). This single-stranded DNA virus can cause high mortality in crickets resulting in colony collapse. AdDV disease outbreaks in cricket mass-rearing can be prevented by either obtaining virus-free crickets or by reducing virus spread. Therefore, insight into viral levels in cricket developmental stages and into viral transmission routes is needed. Viral levels were monitored using quantitative PCR on samples collected 1) simultaneously from different life stages present in the rearing room and 2) weekly from a single rearing container during the successive developmental stages. To study viral tissue tropism and to infer the route of virus transmission, viral levels were measured in cricket tissues and in mated and non-mated adult crickets. Results showed that viral levels increased when developing from nymphs into adults and that unmated individuals had significantly lower viral levels than mated individuals. Furthermore, AdDV was present in every tested tissue and the gut and ovaries of females showed the highest viral levels. Our results suggest that AdDV is both horizontally and vertically transmitted among house crickets and provide relevant information to establish virus-free cricket lines.

## Highlights

• Levels of the Acheta domesticus densovirus (AdDV) increase during house cricket development.
• Mated adult crickets have higher levels of AdDV than unmated ones.
• AdDV is present in the gut as well as the reproductive tissues of adult crickets.
• AdDV is most likely transmitted horizontally as well as vertically among house crickets.

## 1 Introduction

The house cricket, *Acheta domesticus* L., has a long history of commercial production as pet feed and human food source, mainly in Asia and other parts of the world. House crickets have a favorable nutritional profile making them a good candidate as a food ingredient (Montowska et al., 2019). In 2022, partially defatted house cricket powder was authorized by the European Food Safety Authority (Food et al., 2022) for use as food which is sold in the European Union from early 2023 onwards. The increasing interest in house cricket production also increases the importance of stable, consistent and economically viable production of this species. However, house cricket production can be jeopardized by the Acheta domesticus densovirus (AdDV), which is known to cause epidemics and colony collapse in production facilities (Szelei et al., 2011). AdDV is a single-stranded DNA virus from the *Parvoviridae* family (Penzes et al., 2023), belonging to the insect-specific subfamily *Densovirinae*. Viruses belonging to this subfamily are usually highly virulent to their host (Fédière, 2000). Other cricket species, including the commercially produced *Gryllus bimaculatus* and *Gryllodes sigillatus* (Weissman et al., 2012), can also harbour AdDV, that can cause mortality in *G. bimaculatus* (Kim et al., 2024). In *A. domesticus*, AdDV is often present in a covert state, not causing any visible symptoms. Knowledge on which factors trigger an AdDV epidemic is currently limited (Takacs et al., 2023), hampering the possibilities of preventive or curative methods.

A crucial aspect of virus infection dynamics is the transmission route of the virus, since this influences the spread and prevalence of the virus in the population (Chen et al., 2006). The two main types of viral transmission are vertical and horizontal, that can also occur simultaneously (Cory, 2015). Vertical transmission occurs when the parental generation transmits the virus to the offspring, maternally via the egg surface (transovum) or within eggs (transovarial), or paternally via sperm (Chen et al., 2006). Horizontal transmission can take place by ingesting contaminated feed, via cannibalism, via feces, or during mating via contact between males and females (sexual transmission) (Maciel-Vergara and Ros, 2017; Huger, 1985; Knell and Webberley, 2004). House cricket rearing facilities are an ideal environment for virus transmission, with a high cricket density resulting in frequent contact between individuals, feed and feces (Duffield et al., 2021). Current knowledge on AdDV transmission in crickets is limited and a better understanding of virus transmission will help to optimize rearing strategies to prevent transmission and consequently viral outbreaks. Viral levels could be reduced by limiting horizontal transmission using disinfection procedures. Alternatively, if vertical transmission can be prevented, virus-free cricket-breeding stocks can be created by selecting uninfected individuals via non-destructive sampling such as screening feces. Additionally, densoviruses are known to be capable of replicating in different insect host tissues including the fat body, gut and ovaries (Tijssen et al., 2016). Measuring viral levels in different tissues can give clues about the transmission mode: with horizontal transmission, high viral levels are expected in the gut since this is the organ through which the virus enters the body after oral infection, while in case of vertical transmission, high viral levels are expected in the reproductive tissues. To enhance our understanding of AdDV abundance and transmission, we evaluated viral levels in different tissues of crickets, including mated and non-mated adults. In addition, we measured viral levels during nymphal development and in the adult stage to apprehend fluctuations in viral levels during house cricket production.

## 2 Materials and methods

### 2.1 House cricket colony and sampling

The *A. domesticus* colony has been kept at the Laboratory of Entomology, Wageningen University & Research, since 2006 in two lines, the main and the backup line. Crickets freshly hatching on first four days of the week are collected for the so called “main” rearing line, while the freshly hatched crickets from the second three days of the week, are collected for the “backup” line. The lines are only differing in the pinhead collection time, otherwise thy are entirely identical in their conditions. Having the two lines running parallel allowed for duplicate sampling of the given age groups (see below). Approximately, 500 crickets are housed in 40 L plastic containers in a climate chamber at _∼_28 °C with ∼50 % relative humidity (RH) and are provided with chicken feed (Kuiken Opfokmeel, Kasper Fauna Food, Woerden, the Netherlands), carrot slices and water from bird water dispensers. At any given time, six different age groups (Table 1) are simultaneously present, ranging from the youngest instars to fully developed adults with approximately one week of age difference.

**Table 1:** Group numbers with the associated age ranges, average body weight and the samples sizes used in the two types of sampling.

Age group	Age of crickets (days)	Average weight (SD) (mg)	Snapshot sampling # individuals****	Sequential sampling # individuals****
1	0–7*	NA	16	16
2	7–14	9.7 (2)	16	16
3	14–21	47.2(16.6)	16	16
4	21–28	221.5 (76.1)	16	16
5	28–35**	304.7 (80.5)	0	16
6	35–42***	356.8 (75.9)	16	16

*Only pinheads were sampled **Only the last nymphal stage before adulthood was sampled. ***Only adults were sampled. ****Per age group, half of the samples (8) were obtained from the main line, and the other half (8) from the backup line. SD: standard deviation.

Two sampling approaches were used to investigate viral levels in the different lines and age groups, from pinheads to adults. Firstly, a “snapshot” sampling was done. This involved sampling pinheads and nymphs of different ages and adults present in the rearing chamber at a single time point, from both the main and backup line. Five different age groups were sampled in the snapshot sampling (age groups 1, 2, 3, 4 and 6; Table 1). When it was possible to determine the sex (for crickets older than four weeks), equal numbers of males and females were taken. Secondly, a sequential sampling was done from rearing containers that were initiated with recently hatched pinheads (for both the main and backup line) and then subsequently sampled for six consecutive weeks (age groups 1 to 6; Table 1). Samples were chilled, placed in an 1.5 ml Eppendorf tube and stored at −20 °C in PBS buffer until DNA extraction.

### 2.2 Tissue dissections

Tissue dissections were performed on non-mated and mated adults of six weeks old of one line (since neonate hatching). To obtain non-mated adults, five-weeks-old nymphs were collected from the colony and separated by sex. These crickets were kept in incubators under similar conditions as the main rearing until sexual maturity was reached (ovipositor visible in female crickets) at the age of six weeks. To obtain tissues from mated individuals, six-weeks-old crickets were collected from the main rearing colonies when mating and egg deposition were observed. Collected crickets were transferred to incubators but in this case, sexes were kept mixed. Next, all crickets were starved for 48 h by removing the feed source and provided with only a water source prior to dissection.

Dissections were done under a stereomicroscope in sterile phosphate-buffered saline (PBS). For mated and non-mated females, the gut tracts, the ovaries with the eggs and the spermatheca (only for mated females) were collected. For mated and non-mated males, the gut tracts, the testes and the accessory glands were collected. For each tissue type, eight replicates (each consisting of pooled tissues from three individuals) were collected. Samples were stored in PBS solution at −20 °C until DNA extraction.

### 2.3 DNA extraction and quantitative PCR

After the samples were thawed, each sample was homogenized with a sterile pestle in a 1.5 ml Eppendorf® tube, followed by centrifugation at 14,000 rpm for 10 min. The supernatant was used for DNA extraction, to remove the rigid exoskeleton parts. The DNA extraction was done using the Qiagen DNeasy® Blood and Tissue kit (Qiagen, Germany), following the ‘Total insect DNA extraction’ protocol, with sample homogenization described as above. Quality control of the DNA extraction was done using Nanodrop® ND1000 Spectro-photometer (Thermo Fisher Scientific Inc., United States).

A relative quantitative PCR (qPCR) was performed to quantify the AdDV viral levels in the samples. To this aim, a 96 base pair (bp) fragment of the AdDV gene encoding the non-structural protein 2 (NS2) was amplified, using primers reported by Duffield et al. (2021) (Table 1). As a host reference gene, 199 bp of the *Acheta domesticus elongation factor 1 alpha* gene (*ef1a*; Genbank access nr. EU414685.2) was amplified (Table 2). Reactions were performed with SYBR™ Select Master Mix (Thermo Fisher Scientific Inc., United States), as described by Takacs et al. (2023).

**Table 2:** Primers used in qPCR.

Name	Reference	Primer sequence 5′ to 3′ (forward / reverse)	Amplicon size (bp)	Gene target
EF1a	This study	GGAAATCAAGAAGGAAGTCAGC/ GGCATCCAAAGCCTCAATAAG	199	*A. domesticus* elongation factor 1 alpha
NS2	Duffield et al., 2021	GCGAGCAATCCCGACTACTA/ CGCGTTGTTGATGTCCTTCC	96	AdDV non-structural protein 2

### 2.4 Data analyses

The normalized viral levels of AdDV were calculated by the following formula:

$$\text{lo}g_{10}\left(E^{-\text{Ct}\left(\text{AdDV}\right)}/E^{-\text{Ct}\left(\text{EF}1a\right)}\right)$$

in which E represents the amplification efficiency, which equals two in case of 100 % efficiency, and Ct represents the threshold cycle value of the qPCR amplification either for the viral (AdDV) or the host (EF1a) targets (Hernandez-Pelegrin et al., 2022). The amplification efficiency was calculated for every plate and primer pair and were in the range of 95–105 %. This was confirmed via dilution series to obtain a standard curve.

Next, the data were subjected to a Shapiro-Wilk test of normality (Shapiro and Wilk, 1965) and a Levene test to assess the homogeneity of variances (Levene, 1960). For datasets that met the assumptions of normality and homogeneity of variance (Table S1), a two-way analysis of variance (ANOVA) was used to test the effect of the variables and their interaction. The Tukey's HSD (honestly significant difference) test was used for pairwise comparisons. If data did not meet the abovementioned assumptions (Table S1), the effect of the main factors and their interaction on normalized viral levels was analyzed using a linear models (lms) and Type I sums of squares ANOVA. The different lms were compared based on the second-order Akaike information criterion (AIC) values and the best fitting models with the lowest AIC were selected (Burnham and Anderson, 2004). If the main factors or their interactions had a significant effect, pairwise comparisons were carried out with the R package ‘emmeans’, version 1.8.4–1 (Searle et al., 1980). Analyses and visualization were carried out using R, version 4.2.2 (2022–10–31) (R Core Team 2020) and ggplot2 (Wickham, 2016) in R Studio.

## 3 Results

### 3.1 Viral levels vary throughout nymphal development into the adult stage

**Fig. 1:**
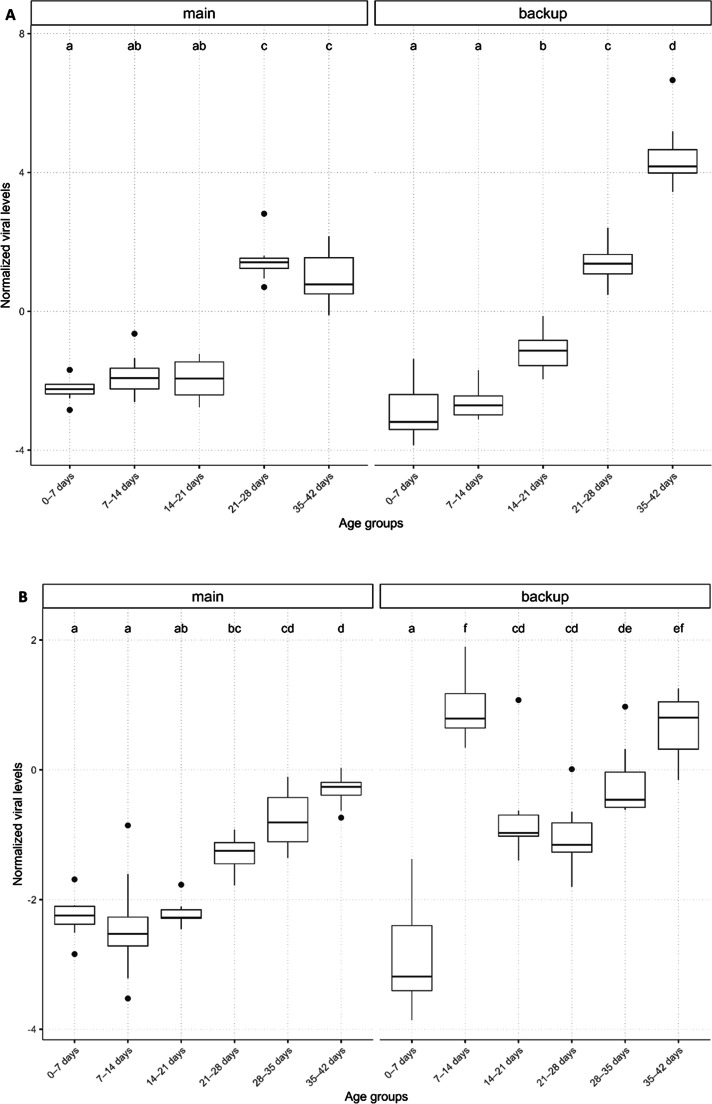
Normalized viral (DNA) levels (relative to the host EF1a gene) for each age groups of crickets sampled from the rearing using snapshot sampling (A) and sequential sampling (B). The Y-axis represents the normalized viral levels, while the X-axis represents the age groups of the crickets (Table 1). The headers main and backup indicate the two different cricket lines that were tested. The boxplots show the median (horizontal line), the lower and upper quartiles; vertical lines extend to the lowest and highest values. Dots represent outliers, age groups that have no letters in common differ significantly*.*

Snapshot sampling was used to assess the normalized viral levels of two different cricket lines in five age groups (Table 1). The sequential (Type I) ANOVA indicated a significant interaction between the cricket line and the age group (F_4,70_ = 26.18, *p* = 2.74e-13) (Table S1). We observed significantly higher viral levels for the older age groups (21–28 days and 35–42 days) from both lines compared to the younger age groups (all *p* < 0.0001, Table S2 and Fig. 1A). The first two age groups (0–7 days and 7–14 days) from both lines, as well as the third age group (14–21 days) of the main line, all had similar low viral levels (Fig. 1A, Table S2). Viral levels were the highest for crickets of 35–42 days old of the backup line and were significantly different compared to viral levels of 21–28 days old crickets from both lines (both *p* < 0.0001, Table S2). For the sequential sampling method, the selected rearing containers were sampled weekly for six consecutive weeks (representing six age groups, Table 1). Similarly, statistical tests showed a significant interaction between the cricket line and the age group (F_5,84_ = 22.91, *p* = 1.95e-14). The lowest median viral level was found in the youngest crickets sampled between 0–7 days, which was significantly lower than viral levels found in crickets of the main line that were 21 days and older (*p* < 0.0001 for all comparisons) (Fig. 1B, Table S3). In the backup line, the crickets that were between 7 and 14 days old showed a high viral level, which caused this group to have a viral level similar to that of adults from the same line (Table S3). Overall, a consistent increase in viral levels was observed towards adulthood of the crickets for both cricket lines and for both sampling approaches.

### 3.2 A major effect of mating on viral levels in individual crickets

**Fig. 2:**
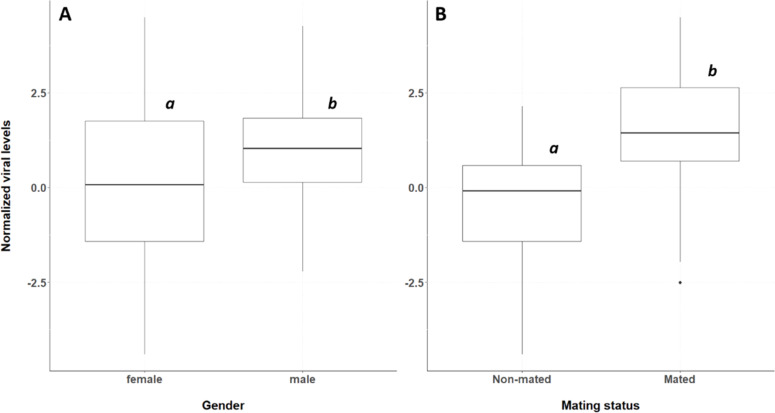
Normalized viral (DNA) levels (relative to the host EF1a gene) of males and females (A) and of non-mated and mated adult crickets (B). The Y-axis represents the normalized viral levels, while sex and mating status are indicated along the X-axis. The boxplots show the median (horizontal line), the lower and upper quartiles; vertical lines extend to the lowest and highest values. Age groups that have no letters in common differ significantly.

Both statistical tests showed significant effects of sex (F_1,86_ = 6.43, *p* = 0.013) and mating status (F_1,85_ = 36.57, *p* = 3.93e-8) on the normalized viral levels in adult crickets of 35–42 days old. The best fitting model did not show a significant interaction term between mating status and sex (F_1,84_ = 2.79, *p* = 0.0988) (Table S1). More specifically, male crickets had significantly higher viral levels than female crickets (*p* = 0.0014) (Fig. 2A). The viral levels of mated individuals were also significantly higher compared to non-mated individuals (*p* < 0.0001) (Fig. 2B).

### 3.3 Viral tissue tropism in the different sexes

**Fig. 3:**
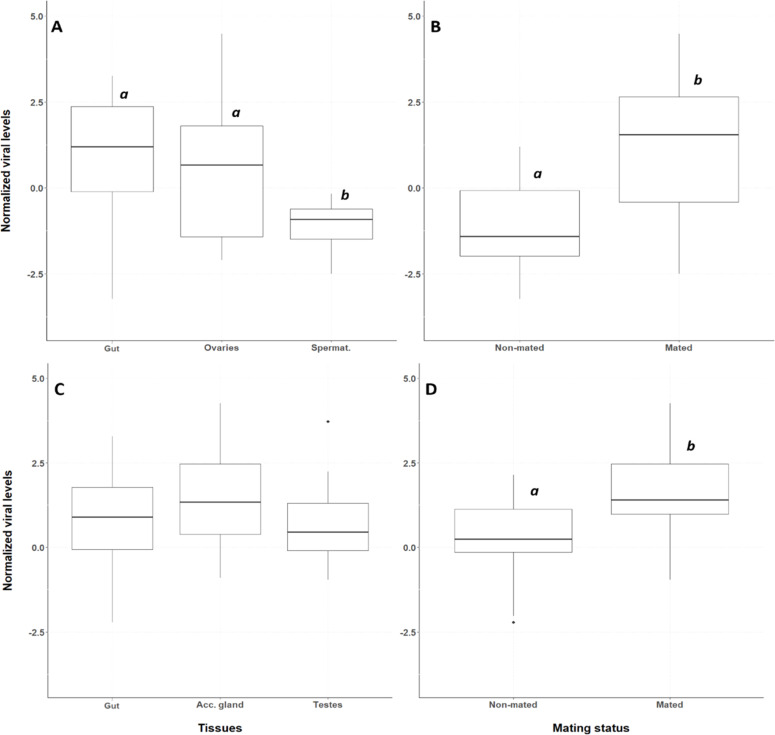
Normalized viral (DNA) levels (relative to the host EF1a gene) in tissues of female house crickets (A) between non-mated and mated females (B) and in tissues of male house crickets (C) and between non-mated and mated males (D). The Y-axis represents the normalized viral levels, while the X-axis represents the different tissues (A, C) (‘Spermat’ refers to spermatheca) and the mating status (B, D) (‘Acc. gland’ refers to accessory gland). The boxplots show the median (horizontal line), the lower and upper quartiles; vertical lines extend to the lowest and highest values. Boxplots that have no letters in common differ significantly between the tissues (A, C) and between mated and non-mated females (B) or males (D).

In female house crickets, AdDV was detected in all tested tissues. Both the mating status and the tissue type had a significant effect on the viral levels (F₁,₃₅ = 37.86, *p* = 4.86e-7 and F_2_,₃₅ = 18.87, *p* = 2.76e-6, respectively), and there was no interaction between these factors (F₁,₃₅ = 0.026, *p* = 0.872) (Fig. 3). Among the tissues, the guts and the ovaries had similar viral levels (*p* = 0.9144), which were significantly higher than found in the spermatheca (both *p* < 0.0001) (Fig. 3A). Mated female crickets had significantly higher viral levels than non-mated ones (*p* < 0.0001) (Fig. 3B). In male house crickets, all the tested tissues were positive for the presence of AdDV, however, there was no effect of tissue type on the viral levels (F_2_,_42_ = 2.49, *p* = 0.095) (Fig. 3C). Only mating status had a significant effect on viral levels (F_1_,_42_ = 18.35, *p* = 0.0001), with mated males having significantly higher viral levels than non-mated ones (*p* = 0.0001) (Fig. 3D).

## 4 Discussion

In this study, we investigated viral levels during house cricket nymphal development into the adult stage and viral tissue tropism, to infer possible viral transmission routes. We found that AdDV levels generally increased from the nymphal to adult stage, although levels were affected by the interaction between line and cricket age. Younger crickets had lower viral levels, with the exception of 7–14 days old crickets in the sequential sampling experiment, possibly due to a batch effect or local outbreak. Viral levels were higher in males compared to females, and higher in mated adults compared to unmated adults. Furthermore, AdDV was present in every tested tissue of the adult crickets and significant differences in viral levels were found between the different tissues of females, with higher levels in the gut and ovaries compared to the spermatheca. Detailed knowledge on these aspects will help to identify possible interference points with the aim to develop colonies low in viral levels, or completely free of viruses.

Viral levels showed to increase during cricket nymphal development into the adult stage, with the highest levels found in the adult stage. This was similar to a recent study where viral loads were found to increase with cricket aging (Boykin et al., 2025). In addition, the virus was detected in the gut, indicating that the virus is likely transmitted horizontally. Since nymphs grow in size while the available surface area in the rearing container remains the same, the chance of physical interactions (*e.g.*, direct contact, mating or cannibalism) between individuals increases, leading to increased probability of horizontal transmission of AdDV. Furthermore, exposure to virus is enhanced by increasing contact of the larger crickets with contaminated surfaces, feed and feces. In addition, stress due to physical interactions can have a major impact on the cricket’s immune defense, which might lead to increased viral levels. An earlier publication on AdDV mentioned that mortality due to AdDV is the most prevalent in the late instars of house crickets (Szelei et al., 2011). Our findings are in line with this observation and showed an increase in viral levels during cricket development (Szelei et al., 2011). Szelei et al. (2011) found AdDV to be detectable in the air filters of the climate room, which suggests fast viral spread via the air throughout the entire facility. The above-mentioned scenarios suggest horizontal transmission of the virus and highlight the difficulty of eradicating the virus.

The presence of the virus in the adult reproductive tissues suggests that the virus can also be vertically transmitted. Further work should focus on the role of eggs or sperm in the vertical transmission. If the virus is transmitted on the surface of the eggs, decontamination treatments, like a controlled dosage of UV light, could be tested (Virto et al., 2013). Preventing vertical transmission from the parental generation to the offspring, or selecting virus-free offspring if vertical transmission is <100 %, would allow the establishment of virus-free breeding stocks of house crickets.

The increase of AdDV levels happens simultaneously with sexual maturation and mating. We found that mated house crickets of both sexes exhibited a higher viral level compared to non-mated individuals. Furthermore, it was previously reported that mated female house crickets generated a lower immune response compared to non-mated females upon receiving an immune challenge (Bascunan-Garcia et al., 2010). This might explain our results of mated females having a significantly higher viral level compared to non-mated ones. Because egg production is a relatively costly process for female insects (Schwenke et al., 2016), and known to reduce the available resources for the immune system (Short and Lazzaro, 2010), a shift in resource allocation may pave the way for pathogen levels to increase. How mating exactly affects viral levels in crickets warrants further investigation.

Sperm of insects can be utilized by viruses to ‘hitchhike’ to females (Mao et al., 2019). After mating, female insects store the spermatophore from the males in the spermatheca. This organ is also responsible for maintaining and releasing the sperm to fertilize the eggs (Pascini and Martins, 2017). The viral level of the spermathecae collected from all mated females (confirming their mated status) was lower than in the other female tissues. Taking into account the high viral levels found in the testes and accessory glands of male crickets, the low levels found in the spermatheca were unexpected. However, as sperm was not tested separately, it cannot be concluded if either the testes or the sperm itself exhibit high viral levels. The role of the male accessory glands is to facilitate transport and maturation for the sperm cells, and to provide them with nourishment essential for sperm viability (Happ, 1984). Viruses could utilize the accessory glands for the hitchhiking (Mao et al., 2019) with a higher probability of vertical transmission in mated than in non-mated males, which correlates with the higher viral levels found in the mated crickets. However, the virus is possibly present in all cricket tissues, and therefore its presence in the reproductive tissues cannot be directly correlated to a vertical transmission route of AdDV. Additional experiments including crossings with uninfected crickets would help to further elucidate this transmission route.

House cricket colonies are likely to harbor an AdDV covert infection, which can be triggered to an overt, lethal infection wiping out complete mass-rearing populations (Szelei et al., 2011; Weissman et al., 2012). Therefore, information on viral levels and transmission during cricket development is indispensable to reduce or eradicate AdDV in colonies. We detected the virus in all dissected tissues, suggesting horizontal and vertical transmission, and observed that the viral level steadily increased over time during the life cycle, reaching the highest values in adult crickets. This emphasizes that measures should be aimed at reducing AdDV to manageable levels, for example by separating offspring from the adult generation to reduce virus transmission. To further prevent disease outbreaks, a more in-depth understanding of AdDV-cricket interactions and of AdDV transmission is needed (Takacs et al., 2023), which will ultimately aid the development of preventive and/or curative methods (Eilenberg et al., 2015).

## Data availability

All data are provided in the supplementary materials.

## Funding

This project has received funding from the European Union Horizon 2020 research and innovation program, under the Marie Skłodowska-Curie grant agreement 859850. V.I.D.R. is supported by a VIDI grant from the Dutch Research Council (NWO; VI.Vidi.192.041).

## Author contributions

JT, AB, JJAvL, and VIDR conceived the study and designed experiments. JT performed the experimental work. JT and AB performed data analysis and visualisation and wrote the original draft. JT, AB, ABJ, JJAvL and VIDR edited and reviewed the final draft.

## Declaration of competing interest

The authors declare the following financial interests/personal relationships which may be considered as potential competing interests: Jozsef Takacs reports financial support was provided by European Union. Vera I.D. Ros reports financial support was provided by Dutch Research Council.

## Appendix Supplementary materials

Image, application 1

Image, application 2

Image, application 3
