# Supplementary material for: Tissue tropism and viral levels of Acheta domesticus densovirus throughout the house cricket production
Source: Curr Res Insect Sci. 2025 Dec 20;9:100121. doi: 10.1016/j.cris.2025.100121 (PMC12814073; doi:10.1016/j.cris.2025.100121)
Supplement: Supplementary file 3 [file mmc3.docx]

**Supplementary tables**

| Experiment | Response (y) | Model | Formula | Distribution | Post-hoc testing | Factor | Df | Deviance/  Sum Squares | Residual deviance/ Mean Squares | F | Pr(>F) | Sign.^¥^ |
| --- | --- | --- | --- | --- | --- | --- | --- | --- | --- | --- | --- | --- |
| Viral levels throughout the cricket development – snapshot sampling | Viral level | glm | y ~ Cricket Age * Line | Gaussian | emmeans | Cricket Age | 4 | 356.7 | 89.91 | 187.77 | < 2.2e-16 | *** |
|  |  |  |  |  |  | Line | 1 | 6.93 | 82.98 | 14.59 | 2.9e-4 | *** |
|  |  |  |  |  |  | Cricket Age*Line | 4 | 49.73 | 33.24 | 26.18 | 2.7e-13 | *** |
| Viral levels throughout the cricket development – sequential sampling | Viral level | glm | y ~ Cricket Age * Line | Gaussian | emmeans | Cricket Age | 5 | 70.27 | 86.96 | 44.73 | 2.2e-16 | *** |
|  |  |  |  |  |  | Line | 1 | 24.58 | 62.38 | 78.25 | 1.21e-13 | *** |
|  |  |  |  |  |  | Cricket Age*Line | 5 | 35.99 | 26.39 | 22.91 | 1.95e-14 | *** |
| Effect of mating and sex on the viral levels in individual crickets | Viral level | glm | y ~ Sex*Mating Status | Gaussian | emmeans | Sex | 1 | 14.39 | 276.0 | 6.43 | 0.013 | ** |
|  |  |  |  |  |  | Mating Status | 1 | 81.82 | 194.18 | 36.57 | 3.93e-8 | *** |
|  |  |  |  |  |  | Sex*Mating Status | 1 | 6.23 | 187.95 | 2.79 | 0.0988 |  |
| Viral tissue tropism in females | Viral level | ANOVA – two way | y ~ Mating Status * Tissue | normal | Tukey's HSD | Mating Status | 1 | 62.27 | 62.27 | 37.86 | 4.86e-7 | *** |
|  |  |  |  |  |  | Tissue | 2 | 62.07 | 31.03 | 18.87 | 2.76e-6 | *** |
|  |  |  |  |  |  | Mating Status * Tissue | 1 | 0.04 | 0.04 | 0.026 | 0.872 |  |
|  |  |  |  |  |  | Residuals | 35 | 57.57 | 1.64 |  |  |  |
| Viral tissue tropism in males | Viral level | ANOVA – two way | y ~ Mating Status * Tissue | normal | Tukey's HSD | Mating Status | 1 | 25.78 | 25.78 | 18.35 | 0.0001 | *** |
|  |  |  |  |  |  | Tissue | 2 | 7.00 | 3.50 | 2.49 | 0.095 |  |
|  |  |  |  |  |  | Mating Status * Tissue | 2 | 2.26 | 1.13 | 0.803 | 0.454 |  |
|  |  |  |  |  |  | Residuals | 42 | 59.01 | 1.41 |  |  |  |

|  |  |  |  |  |  |
| --- | --- | --- | --- | --- | --- |

*Table S1. Different data analysis methods used for the different datasets.* ^¥^Sign. means the significance of the p-value with asterisks indicating the level of significance: * (p < 0.05), ** (p<0.01), *** (p<0.001).

|  | **0–7 days (backup)** | **0–7 days (main)** | **7–14 days (backup)** | **7–14 days (main)** | **14–21 days (backup)** | **14–21 days (main)** | **21–28 days (backup)** | **21–28 days (main)** | **35–42 days (backup)** | **35–42 days (main)** |
| --- | --- | --- | --- | --- | --- | --- | --- | --- | --- | --- |
| **0–7 days (backup)** | NA | 0.9891 | 1.000 | 0.2172 | 0.0002 | 0.4110 | 0.0000 | 0.000 | 0 | 0.0000 |
| **0–7 days (main)** | 0.9891 | NA | 1.000 | 1.000 | 0.0642 | 1.000 | 0.0000 | 0.000 | 0 | 0.0000 |
| **7–14 days (backup)** | 1.000 | 1.000 | NA | 0.7021 | 0.0017 | 0.9053 | 0.0000 | 0.000 | 0 | 0.0000 |
| **7–14 days (main)** | 0.2172 | 1.000 | 0.7021 | NA | 0.8134 | 10.000 | 0.0000 | 0.000 | 0 | 0.0000 |
| **14–21 days (backup)** | 0.0002 | 0.0642 | 0.0017 | 0.8134 | NA | 0.5687 | 0.0000 | 0.000 | 0 | 0.0000 |
| **14–21 days (main)** | 0.4110 | 1.000 | 0.9053 | 1.000 | 0.5687 | NA | 0.0000 | 0.000 | 0 | 0.0000 |
| **21–28 days (backup)** | 0.0000 | 0.0000 | 0.0000 | 0.0000 | 0.0000 | 0.0000 | NA | 1.000 | 0 | 0.9999 |
| **21–28 days (main)** | 0.0000 | 0.0000 | 0.0000 | 0.0000 | 0.0000 | 0.0000 | 1.000 | NA | 0 | 0.9980 |
| **35–42 days (backup)** | 0.0000 | 0.0000 | 0.0000 | 0.0000 | 0.0000 | 0.0000 | 0.0000 | 0.000 | NA | 0.0000 |
| **35–42 days (main)** | 0.0000 | 0.0000 | 0.0000 | 0.0000 | 0.0000 | 0.0000 | 0.9999 | 0.998 | 0 | NA |

*Table S2. P values for pairwise comparisons of the snapshot sampling viral level data.*

|  | **0–7 days (backup)** | **0–7 days (main)** | **7–14 days (backup)** | **7–14 days (main)** | **14–21 days (backup)** | **14–21 days (main)** | **21–28 days (backup)** | **21–28 days (main)** | **28–35 days (backup)** | **28–35 days (main)** | **35–42 days (backup)** | **35–42 days (main)** |
| --- | --- | --- | --- | --- | --- | --- | --- | --- | --- | --- | --- | --- |
| **0–7 days (backup)** | NA | 0.9359 | 0.0000 | 0.9999 | 0.0000 | 0.8276 | 0.0000 | 0.0000 | 0.0000 | 0.0000 | 0.0000 | 0.0000 |
| **0–7 days (main)** | 0.9359 | NA | 0.0000 | 1.000 | 0.0000 | 1.000 | 0.0023 | 0.0596 | 0.0000 | 0.0000 | 0.0000 | 0.0000 |
| **7–14 days (backup)** | 0.0000 | 0.0000 | NA | 0.0000 | 0.0000 | 0.0000 | 0.0000 | 0.0000 | 0.0077 | 0.0000 | 1.000 | 0.0019 |
| **7–14 days (main)** | 0.9999 | 1.000 | 0.0000 | NA | 0.0000 | 1.000 | 0.0003 | 0.0095 | 0.0000 | 0.0000 | 0.0000 | 0.0000 |
| **14–21 days (backup)** | 0.0000 | 0.0000 | 0.0000 | 0.0000 | NA | 0.0001 | 1.000 | 0.9330 | 0.9917 | 1.000 | 0.0002 | 1.000 |
| **14–21 days (main)** | 0.8276 | 1.000 | 0.0000 | 1.000 | 0.0001 | NA | 0.0046 | 0.1044 | 0.0000 | 0.0001 | 0.0000 | 0.0000 |
| **21–28 days (backup)** | 0.0000 | 0.0023 | 0.0000 | 0.0003 | 1.000 | 0.0046 | NA | 1.000 | 0.2204 | 1.000 | 0.0000 | 0.5265 |
| **21–28 days (main)** | 0.0000 | 0.0596 | 0.0000 | 0.0095 | 0.9330 | 0.1044 | 1.000 | NA | 0.0118 | 0.9778 | 0.0000 | 0.0436 |
| **28–35 days (backup)** | 0.0000 | 0.0000 | 0.0077 | 0.0000 | 0.9917 | 0.0000 | 0.2204 | 0.0118 | NA | 0.9678 | 0.1358 | 1.000 |
| **28–35 days (main)** | 0.0000 | 0.0000 | 0.0000 | 0.0000 | 1.000 | 0.0001 | 1.000 | 0.9778 | 0.9678 | NA | 0.0001 | 0.9997 |
| **35–42 days (backup)** | 0.0000 | 0.0000 | 1.000 | 0.0000 | 0.0002 | 0.0000 | 0.0000 | 0.0000 | 0.1358 | 0.0001 | NA | 0.0415 |
| **35–42 days (main)** | 0.0000 | 0.0000 | 0.0019 | 0.0000 | 1.000 | 0.0000 | 0.5265 | 0.0436 | 1.000 | 0.9997 | 0.0415 | NA |

*Table S3. P values for pairwise comparisons of the sequential sampling viral level data.*

**Supplementary files**

Supplementary file S1: Raw qPCR data for viral levels across developmental stages

Supplementary file S2: Raw qPCR data for viral levels across tissues
